# New Cultivars of *Galanthus nivalis* in Slovenia

**DOI:** 10.3390/plants13131728

**Published:** 2024-06-22

**Authors:** Jože Bavcon, Blanka Ravnjak

**Affiliations:** Biotechnical Faculty, University Botanic Gardens Ljubljana, 1000 Ljubljana, Slovenia; blanka.ravnjak@bf.uni-lj.si

**Keywords:** *Galanthus nivalis*, intra-species diversity, new cultivars, fragrant snowdrop

## Abstract

In Slovenia there is only one species of common snowdrop (*Galanthus nivalis* L.) that occurs in all four of its phytogeographical regions. Slovenia is located in the center of the distribution of this species. At some sites the subpopulations of snowdrop are common and abundant, but at other sites they may consist of only a few individuals within small populations. *Galanthus nivalis* occurs in a diversity of habitats and individual plants within stands are extremely variable in many of their characteristics. The purpose of this research is to determine the diversity within the species and to identify new stable variants that are interesting for horticultural purposes and use. We examined plants in populations that were the most diverse and isolated individual characteristics from them, which we then cultivated for several years and determined their stability. We found two new varieties that have a very distinctive smell, and one of the new varieties retains its outer perigone leaves completely closed at the end of flowering. The new varieties belong to three different groups: the Skirt group, the Imperial group and the Green group.

## 1. Introduction

The species *Galanthus nivalis* was already described under this name by Linnaeus in his *Species Plantarum* (1753). He described its habitat as: *ad radices Alpinum Veronnae, Tridenti, Viennae* [1]. For the present-day territory of Slovenia, the species is also listed by Scopoli [2,3], noting its habitat as *ad radices montium*. Today, the natural range of *G. nivalis* is estimated to extend from the Pyrenees in the west to western Ukraine in the east. In the north, the species does not grow naturally further north than Paris. and in the south, it is present only in the European portion of Turkey [1]. The intra-species diversity of *G. nivalis* is quite high in its range of distribution, as evidenced by its many early synonyms. The diversity of the species is also evidenced by some old drawings with names prior to the introduction of binomial nomenclature. The common snowdrop was named *Leucoium bulbufum triphyl*, and small and large forms were recognized as *Leucoium bulbufum triphyl Minus* and *Leucoium bulbufum triphyl Maius*. As the drawings show, they were not consistent in the depiction of green leaves. Specifically, the drawings present more leaves than common snowdrop usually has. However, among those drawn there may be varieties depicted that actually do have a greater number of green leaves [4]. The names of both forms of snowdrop survived and were retained when binomial nomenclature was introduced [1]. In his works, Graf (1801–1838) depicted *G. nivalis* as developing two flowers from a single bulb in one of his paintings [5]. Hayek and Markgraf [6] list two described varieties of *G. nivalis*, *G. nivalis biscapus* Beck from 1894 [7], which has two heads, and *G. nivalis maior* Ten., which has 25–50-mm-long outer perigon leaves [8].

Tutin et al. [9] state that the common snowdrop is a highly variable species and that there are several cultivars. Later, varieties of *G. nivalis* were described by different authors as separate species. However, as was subsequently realized, this differences were primarily an expression of morphological variability it turned out later, within the species [1,10]. Examples of historic synonyms include *G. montana* Schur, *G. nivalis* var. *minus* Ten, *G. imperati* and others. All these indicate great diversity within the species, with some characteristics being closely related to certain areas. There has been a tendency to describe individual varieties as new species. The first to be described were the subspecies *G. nivalis* subsp. *reginae-olgae* and *G. nivalis* subsp. *cilicus*. The first is characterized primarily by fall-blooming, and its leaves are often absent at the time. It is described in the Peloponnese. The leaves sprout in the spring and are very narrow, green-blue in colour, with a characteristic central stripe. This subspecies was given species status as *G. reginae-olgae* Orph. and the populations that bloom in spring were given subspecies status as *G. reginae olgae* subsp. *vernalis* Kamari [11]. Their northernmost border of distribution is in Montenegro [12]. *Galanthus nivalis* subsp. *cilicus* was raised to the level of a species named *G. cilicus* Baker, occurring in southern Turkey. In Serbia during the recent revision of genus *Galanthus*, it was found that in addition to the already confirmed species of common snowdrop, some others such as *G. gracilis* Čelak occur there as well, which is recorded as new in the flora of Serbia [13]. Among all species of snowdrop, autumn-blooming species are a rarity. In addition to the already mentioned *G. reginae-olgae*, there is also *G. cilicus* Baker and the species newly described as late as 1994 *G. peshmenii* A. P. Davis & C. D. Brickel [14].

The extraordinary variability of genus *Galanthus* is also evidenced by recent discoveries of new species. *Galanthus koenenianus* Lobin, C. D. Brickel & A. P. Davis was also described in 1994, which blooms from February to March [14]. *Galanthus trojanus* A. P. Davis & N. Őzhtay, which grows in the vicinity of Troy in north-western Turkey, was first recognized in 2001. In 2014, the new species *Galanthus samothracicus* Kit Tan & Biel was described for the area of the North Aegean Islands (Samothrace Island) and in 2019 species *G. bursanus* Zubov, Konca & A. P. Davis [15] was established from the area around the Sea of Marmara in north-western Turkey.

Unlike the genus *Leucojum*, which is considered a polyphyletic group, genus *Galanthus* is considered monophyletic [16,17]. In the genus *Galanthus*, 18 species were described by 2000 [1] and within these as many as 500 varieties were proposed. As many as 80 have been described in species *G. nivalis* alone [18]. Later, with new discoveries, the number of species rose to 24 (World Flora Online), and thousands of cultivars were formed [19,20,21]. The special features of the common snowdrop, at first glance a very common plant, are in the details, which then leads to descriptions of new varieties. It is also one of the first to bloom, which makes the interest in it that much greater [18,22,23,24,25]. Interest in the common snowdrop and in the breeding of new cultivars is mainly present in England, where species *G. nivalis* does not occur naturally. It is planted en masse in gardens, in which other species of this genus appear [22,26,27,28,29,30,31,32,33,34,35]. In addition to these natural species and subspecies, a large number of varieties were found in the gardens and thus many new cultivars were grown. The same applies to North America and Canada, where snowdrops are naturalized and present in large areas. They are also popular in private gardens [19].

In Slovenia, the common snowdrop is a widespread species, and is missing only in some alpine valleys that do not have a touch of the Mediterranean. It actually extends from the sea to the Pannonian Basin, from lower elevations to an altitude of around 1300 m. Slovenia is geographically very diverse and includes four phytogeographical regions: Alpine, Dinaric, sub-Mediterranean and sub-Pannonian [36]. Its terrain and rock composition are also very diverse. All of the above enables a great diversity of the species in Slovenia, which actually lies near the centre of its natural distribution. The first snowdrops bloom in the second or even the first half of December in the Dragonja Valley (coastal region of Slovenia) [37,38,39,40]. However, even there, their blooming is stimulated by the cold. If there is no cold, they bloom around New Year’s. The specimens collected in Dragonja retain the same blooming time in culture in the University Botanic Gardens Ljubljana [40] if the winters are mild and without snow. Their blooming is followed by specimens from Goriška (littoral regions), which bloom at the end of January despite the mild winters. They were observed there in 1752–1753 by Wulfen [39,41,42], who wrote that they bloom at the end of January. Since then, their blooming time has remained the same [39,40]. On the mountain range overlooking the Gulf of Trieste, where the sub-Mediterranean and pre-Alpine climates meet, snow can persist until late April. Because of this, snowdrop still bloom towards the end of March and April and in some years also in May. These specimens retain this late blooming period in culture [40]. In general, the blooming time during very snowy winters can be very long. It extends from the second half of December even to the beginning of May [39]. Phenologically, blooming time has not changed at all in the last 250 years.

Genetically conditioned blooming time and stability of individual varieties have been studied since 2000 also in the University Botanic Gardens Ljubljana. The collection has so far recorded 8458 specimens of species *G. nivalis* collected exclusively from different areas in Slovenia. We found that the more special features a variety has, the worse it is at reproducing naturally. Of course, there are exceptions. Although many stable varieties have already been described as new cultivars based on our collection [39,40,43,44,45,46], in recent years we have discovered new ones in our collection that either remained unnoticed or were dormant for several years in the vegetative stage. In recent years, some of them have bloomed and proved to be interesting and stable new cultivars, which will be described for the first time in this article. The aim of our research is to present some of the stable varieties grown in the ex situ collection of *G. nivalis*, to define them as horticulturally interesting varieties, and to examine whether their variability may depend on geographical location.

## 2. Results

In the case of species *G. nivalis*, the morphology of the flower is the trait that is usually the most variable among specimens, and can therefore be a distinguishing trait between individual varieties and cultivars. Specifically, the number of outer and inner perigon leaves, the length of the perigon leaves, the presence of green colouring of the petals, and the presence of various other modifications (additional petals just below the ovary, equality of outer and inner perigon leaves, double flowers, etc.) can change in the flower. It is on the basis of morphological differences in flowers that individual cultivars of *G. nivalis* are classified into 10 basic groups [20]. Of course, it should be emphasised that representatives of some cultivars may have traits of two or even more groups. In other words, it is not necessary that all traits are characteristic of a particular group. The cultivars we are describing this time belong to three groups: the Green Group, the Imperial Group and the Skirt Group (Table 1).

### 2.1. Green Group

Representatives of the Green Group have an otherwise normal flower shape and a normal number of petals, with the outer perigon leaves having a weak or strong green color. They can be completely coloured, with either larger or smaller spots, or with green stripes [20,44].

#### 2.1.1. *Galanthus nivalis* ‘Dišeči Vilinec’

**Figure 1:**
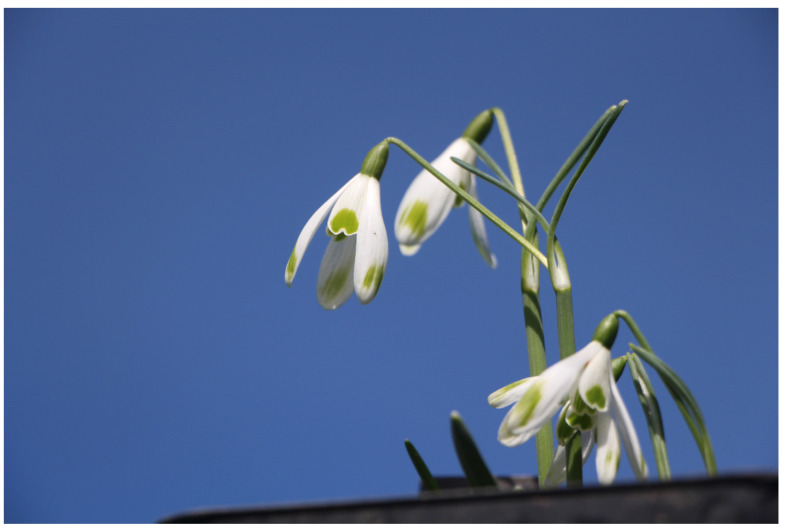
*G. nivalis* ‘Dišeči Vilinec’.

One of the few specimens in which it is possible to detect an obvious pleasant scent are specimens of cultivar *G. nivalis* ‘Dišeči Vilinec’ (Figure 1). The scent of the flowers resembles that of the common lilac (*Syringa vulgaris*). The cultivar is similar in appearance to cultivar ‘La Boheme’ [21], except that the outer perigon leaves of cultivar ‘Dišeči Vilinec’ are rounded, while those of ‘La Boheme’ are more pointed. Also the green stripes on its outer perigon leaves are wider than those of ‘La Boheme’. Individuals of cultivar ‘Dišeči Vilinec’ are late blooming and have 2.5-cm-long flowers as well as outer perigon leaves. There is also a green spot with faded streaks on the lower third of the perigon leaves. The inner perigon leaves are 1.5 cm long and have a broad horseshoe-shaped green spot in the lower third. The ovary is oblong (5–6 mm long). The bracts of the flowers are upright. The flowers are distinctly stalked, giving the quite a pendulous appearance. Flower stalks are 2.5 cm long. Specimens have short green leaves, 4–6 cm long and up to 7 mm wide. Specimens of this cultivar were found in the area of Slovenian Karst.

#### 2.1.2. *Galanthus nivalis* ‘Jakob’s Shell’

**Figure 2:**
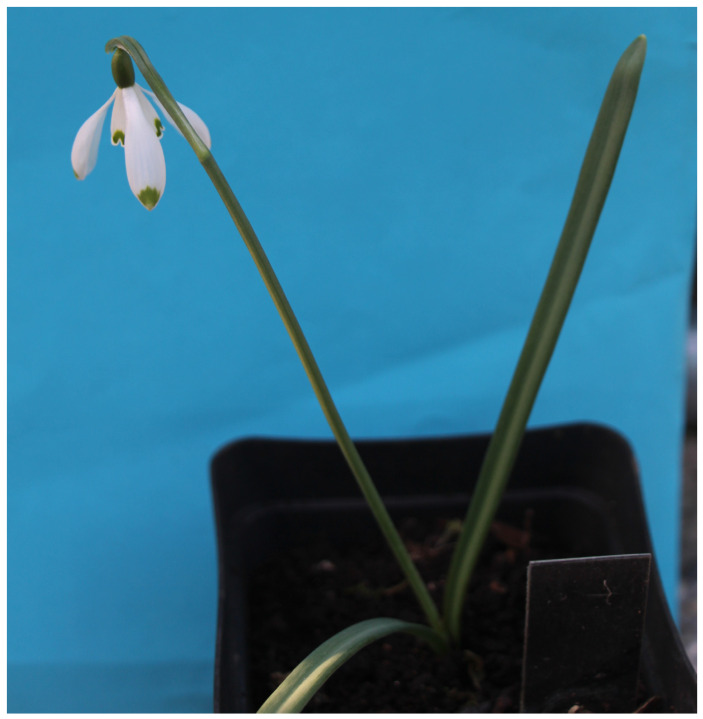
*G. nivalis* ‘Jakob’s Shell’.

The cultivar *G. nivalis* ‘Jakob’s Shell’ is characterized by a larger spot on the tip of the outer perigon leaves, which has the shape of a Jacob’s shell (Figure 2). The spot extends all the way to the tip of the outer perigon leaves. Outer perigon leaves are relatively narrow, up to 3 cm long and 1 cm wide. Inner perigon leaves are more than half the length of the outer ones. They are up to 1.3 cm long and up to 6 mm wide. Flowers bracts are as long as the flower stalk. The ovary is oblong and up to 8 mm long. Its leaves are long, reaching up to 15 cm, and are 7 mm wide. Specimens of the described cultivar were found in western Slovenia. The cultivar belongs to those that bloom sometimes mid-spring. The cultivar is similar to ‘Bella’ [21]. The ‘Jakob’s Shell’ cultivar differs from the ‘Bella’ cultivar in that the green ‘Jakob’s Shell’ pattern on the outer perigon leaves extends all the way to the tip of the leaves, while the ‘Bella’ cultivar has a white tip.

#### 2.1.3. *Galanthus nivalis* ‘Rusalka’

**Figure 3:**
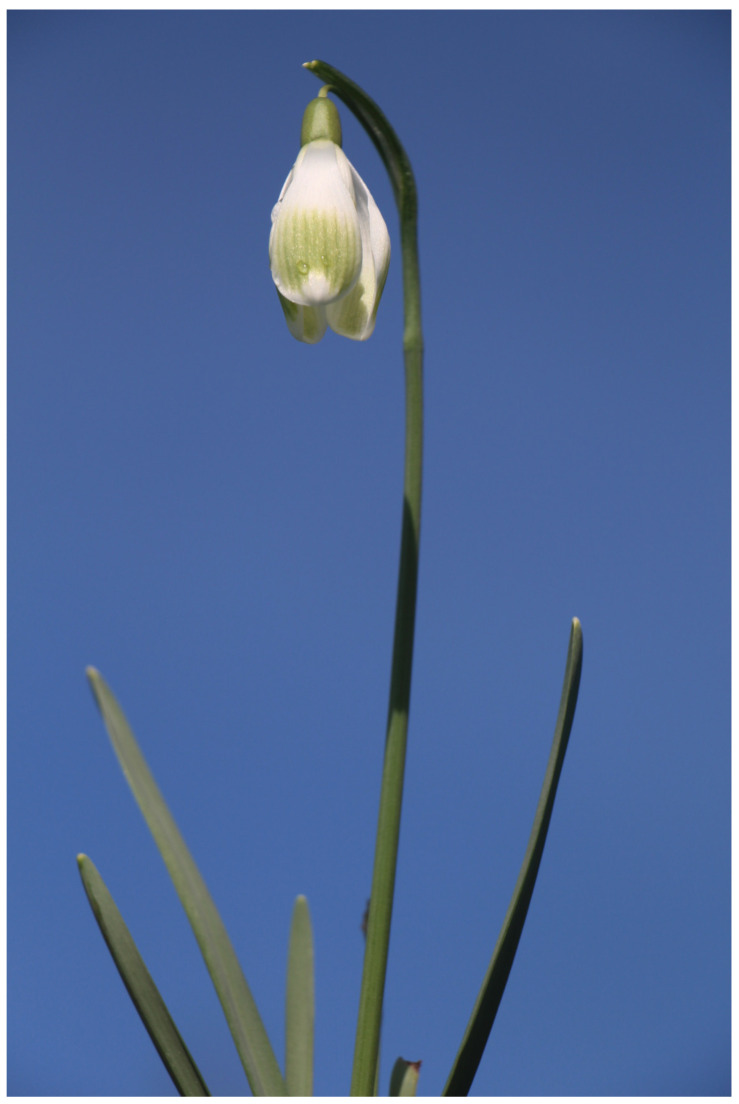
*G. nivalis* ‘Rusalka’.

Specimens of *G. nivalis* ‘Rusalka’ are characterized by very large flowers (Figure 3). The outer perigon leaves can be up to 3.5 cm long, and their width in the widest part is 1.6 cm. Almost halfway up their length, the outer perigon leaves have a pastel green patch with blurred lines. The tips of outer perigon leaves are white. Inner perigon leaves are 1.5 cm long. They also have a large green spot that extends almost half way. The ovary is oblong is 8 mm long and the flower stalks are short. Its leaves are up to 11 cm long and 9 mm wide. *Galanthus nivalis* ‘Rusalka’ has large individuals that can reach 17 cm in height and flower late. Specimens of this cultivar were also found in the area of Slovenian Karst. Cultivar ‘Rusalka’ is somewhat similar to cultivar ‘Ljubljana’ in terms of flower appearance [20,21,39,44,46]. Compared to ‘Ljubljana’, it has more rounded outer perigon leaves with lighter green strips. Also, in ‘Ljubljana’ the tips of the outer perigon leaves are slightly curved inwards, but not by ‘Rusalka’.

#### 2.1.4. *Galanthus nivalis* ‘Senožeče’

**Figure 4:**
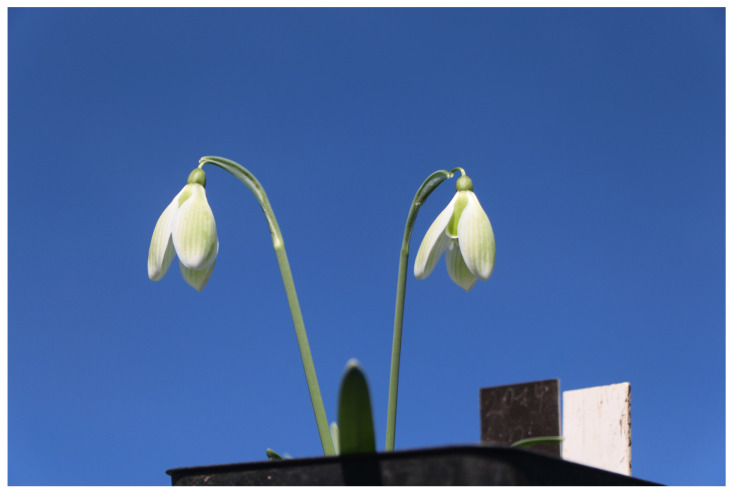
*G. nivalis* ‘Senožeče’.

*G. nivalis* ‘Senožeče’ also belongs to the late flowering varieties, which are also characterised by rather large flowers. Its outer perigon leaves can be up to 2.8 cm long, and their width in the widest part is 1.7 cm. Green streaks are more intensely visible in the green parts of perigon leaves (Figure 4). Outer perigon leaves have a wide ovate shape. Inner perigon leaves are fully green and 1.4 cm long. The ovary is round and up to 4 mm long. Its leaves are up to 9.5 cm long and up to 4 mm wide. The cultivar is somewhat similar to cultivar *G. plicatus* ‘Castle Green Dragon’ [21], except that the described cultivar has more pronounced green stripes on the outer perigon leaves and the inner ones are also more intensely green. The described species also originates from the region of Slovenian Karst.

#### 2.1.5. *Galanthus nivalis* ‘Striped Fragrant’

**Figure 5:**
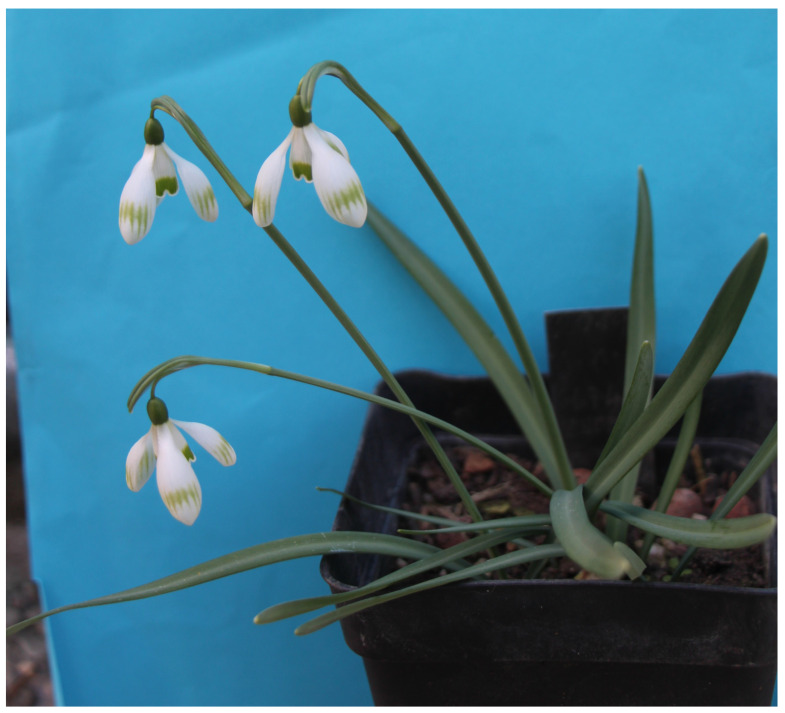
*G. nivalis* ‘Striped Fragrant’.

Another late-flowering cultivar is *G. nivalis* ‘Striped Fragrant’, which is somewhat similar to ‘Carpathian Viridapicis’ [21]. It differs from it in that it smells distinctly and that the green stripes on the outer perigon leaves are less strong coloured that in ‘Carpathian Viridapicis’. *Galanthus nivalis* ‘Striped Fragrant’ is characterized by fragrant flowers with an aroma of common lilac, which is slightly more pronounced than in the cultivar *G. nivalis* ‘Dišeči Vilinec’ (Figure 5). This variety also belongs to those that have large flowers within species *G. nivalis*. The length of the outer perigon leaves reaches 2.8 cm, and the width 1.1 cm. The shape of the outer perigon leaves is spatulate, and a little below the lower half there are green stripes that extend across the entire width of the leaf. Along the length, stripes reach only the lower third of the outer perigon leaves. Inner perigon leaves are up to 1.1 cm long and up to 5 mm wide. A green spot extends almost halfway across their entire width. The ovary is oval and up to 5 mm long. The flower bract arches over the flower stalk. Leaves are up to 10 cm long and up to 6 mm wide. Specimens of this cultivar were also found in nature in Slovenian Karst.

### 2.2. Imperial Group

Specimens with an otherwise normal number of petals, but with inner perigon leaves significantly shorter than the outer ones, belong to the Imperial Group. There is no green coloration or green spots on the outer perigon leaves, and the inner circle of perigon leaves has one green mark [20,39].

#### *Galanthus nivalis* ‘Cardinal’s Hat’

**Figure 6:**
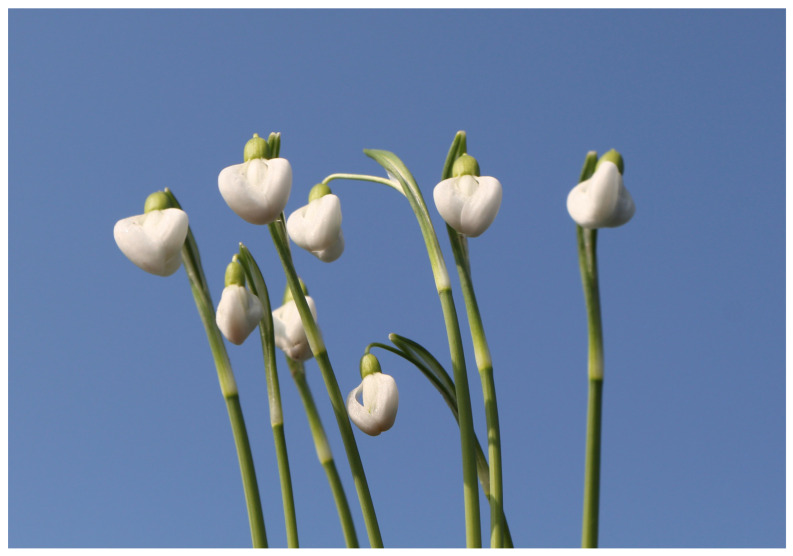
*G. nivalis* ‘Cardinal’s Hat’.

Cultivar *G. nivalis* ‘Cardinal’s Hat’ is late blooming and somewhat resembles *G. plicatus* ‘Diggory’ in terms of flower shape [18,20,21,47], except that the flowers are significantly smaller and the outer perigon leaves are never fully opened (Figure 6). A distinctive feature of *G. nivalis* ‘Cardinal’s Hat’ is that the ends of the outer perigon leaves touch throughout flowering and never open the entrance to the inner perigon leaves. From the point of view of pollinators, access to the stamens is therefore not possible and the flower is self-pollinated. While the outer perigon leaves are 15 mm long and 4 mm wide, the inner perigon leaves are 10 mm long and 5 mm wide. The shape of the outer perigon leaves is distinctly spatulate with a much narrower part near the ovary. They are distinctly concave and resemble the upper lip of the Lamiaceae family in terms of shape. The green pattern on the inner perigon leaves is V-shaped. The ovary is almost round, 3 mm long and a 3–4 mm wide. The bract of the flower is shorter than the stalk. Its leaves are up to 7 cm long and 5–6 mm wide. Unlike the group to which this cultivar belongs, specimens of cultivar *G. nivalis* ‘Cardinal’s Hat’ do not have significantly shorter inner perigon leaves, but they are more or less the same length as in normal specimens of *G. nivalis*. This cultivar is long blooming. Individual specimens grow very upright and are only slightly overhanging. In culture, they multiply very well by bulb division. Specimens of the described cultivar were found on a grassy slope in eastern Slovenia.

### 2.3. Skirt Group

The main characteristic of the group is that the inner perigone leaves are the same length or just a little shorter than the outer ones and that they are also similar in shape. Any additional green or yellow coloration is not present in the outer perigon leaves [20].

#### *Galanthus nivalis* ‘Soldanelca’

**Figure 7:**
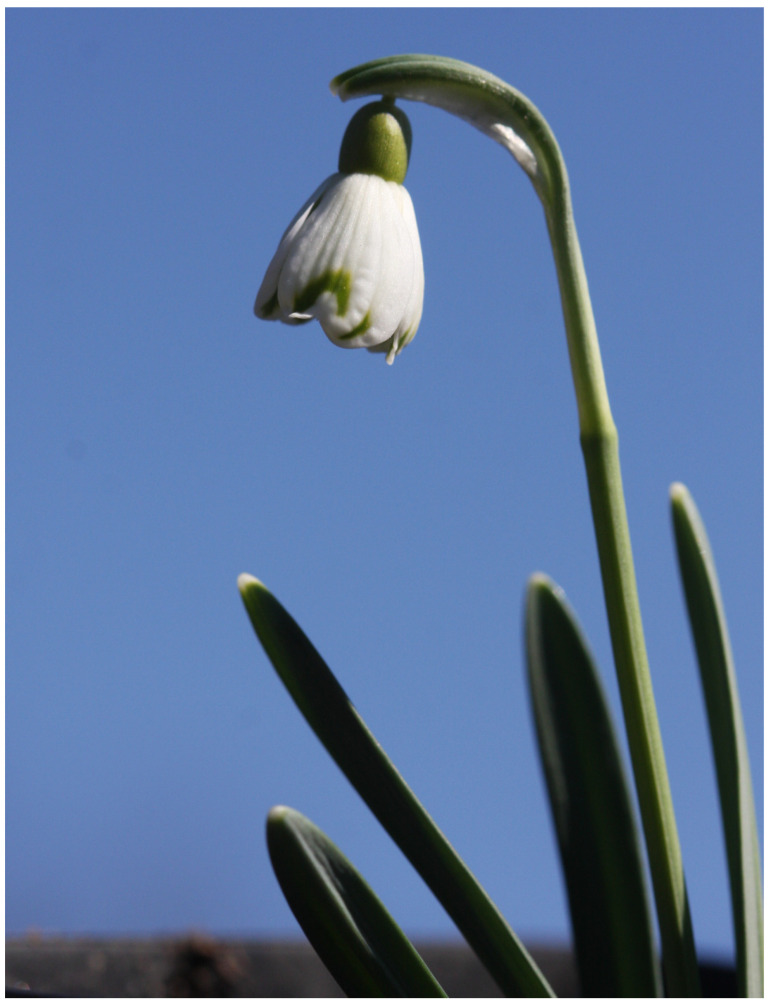
*G. nivalis* ‘Soldanelca’.

The special feature of cultivar *G. nivalis* ‘Soldanelca’ is that the outer perigon leaves have the same shape as the inner perigon leaves (Figure 7). Its outer perigon leaves have a slight notch at the end and a spot in the shape of the letter V. The outer perigon leaves are 1.5 cm long and 8 mm wide. Its inner perigon leaves are about 2 mm shorter than the outer ones. The ovary is oval and up to 4 mm long. Leaves are up to 5 cm long and up to 5 mm wide. Individual specimens are very upright. In terms of appearance, the plant somewhat resembles *Soldanella minima*.

## 3. Discussion

The intra-species diversity of local populations of the common snowdrop varies in different parts of Slovenia. In *Mala flora Slovenije* (Little Flora of Slovenia) [48] we find a description: “Perennial with a one-flowered stem. Two basal leaves, included in a cylindrical scarious sheath. The flowers are nodding, with a bract made of 2 interwoven leaves and the head is fleshy.” Of course, all this is true for normal specimens. However, within such numerous local populations as in Slovenia, we find very interesting deviations from this description in various areas. In some areas the diversity is greater, in others less. In some places, the differences vary more in the shape of the flower, specifically in the length and shape of the outer perigon leaves. Elsewhere, in addition to these variations, green shades appear on the outer perigon leaves, or the number of perigon leaves changes. In some locations, specimens were found with only the outer perigon leaves, which, again, are not uniformly shaped everywhere. If only around 80 varieties of the common snowdrop were known until 2001 [18], in the following decades this number increased tremendously [19,21], as we also confirmed with intensive research in the populations in Slovenia [40].

After more than two decades of intensive research and monitoring of various deviating specimens in culture [38,40,43,45,49,50,51], it turned out that all deviations from the normal form, which we originally considered unstable [43,44], are possible. It is true that some varieties are more stable than others. After so many years of observations of individual varieties in culture and in nature, we can determine with increasing reliability whether the form is stable, perhaps only partially stable, or unstable. Nevertheless, it should be emphasised that just about any form, perhaps even an unusual one, can turn out to be genetically stable. Some unusual shapes are otherwise rare and the number of their repetitions is very small, if there are any repetitions at all. During our fieldwork, we found that individual shapes are found only on some sites, and in intervals of several years. It is only exceptionally that one of the shapes is found at a site with a fairly uniform population. After many years of research, we can confirm for the majority of special varieties of *G. nivalis* that they are fixed to specific sites with an apparently sufficiently large genetic potential, which allows specific morphological traits to be expressed there every few years [40]. At the same time, the fact that this often happens on sites where the population is not even very numerous is also surprising. Sometimes these are numerically small or marginal populations, which can be genetically very diverse [39,40].

The Gaussian curve of species *G. nivalis* is indeed very simple at first glance, as no large deviations are observed. In particular, this is often reflected in very numerous populations in meadows, on the outskirts of forests and in some places in riparian woodland. Mostly, it is only a matter of variations in the internal pattern of perigon leaves and also somewhat in the size of the flower [43,44]. Exceptions on sites with very large populations are rare here [39]. In other locations, specimens of the same population may have quite a bit of diversity with a wider range, but the frequency of individual deviations in such a large population is relatively [40]. What is surprising is the fact that the frequency of individual relatively large deviations often occurs in smaller or even very small populations. Many times, different authors state that the fragmentation of habitats into small local populations leads to a loss of diversity [52,53,54] which is not always necessarily true [55,56]. But our observations in nature indicate that small and numerically weaker populations on the edges or in very heterogeneous habitats, along roads, roadsides, river banks, are often significantly more diverse than much more numerous homogeneous populations on large areas. These often contain more special traits than large compact and homogeneous populations in meadows, riparian woodland and forests. In these large populations, it is quite surprising that the diversity is often very low regardless of the population size [39,40,43,44]. This is not only the case for species *Galanthus nivalis* [44], as a similar conclusion was reaches for species *Cyclamen purpurascens* [44,51,57], genus *Crocus* [38,45], and genus *Helleborus* [38,39,42]. In all these species and genera, as well as in some other studied species in nature [39,58,59,60], the rule that marginal parts of populations are often more diverse is mostly confirmed. Especially where there is a mixture of habitats such as forest–meadow, hedges–meadow, rocky areas in the transition to shallow or deeper ground, etc. [37,38,44,61]. Transitions between individual phytogeographic areas, or areas where their influences mix, are very important [39,40,44,58,61]. If there is also a mixture of growing conditions in these area, i.e., a very heterogeneous habitat, then the possibility of diversity appearing is significantly greater than in the case of a very large local population in very homogeneous conditions. This could be explained by the fact that the heterogeneous habitat conditions create greater pressure on the local population. This leads to earlier splitting of genes and the expression of individual special features. Although the theory of population genetics predicts a reduced diversity of small populations as a result of genetic drift and inbreeding [62,63,64,65], this is in many cases not true in studies of species *G. nivalis* in nature. The opposite is often true, as smaller populations are actually more diverse. This is contrary to the stated facts [66] that small populations could lose individual alleles due to adaptation to a changed environment. An increased level of homozygosity is also the result of inbreeding, which causes pollination and fertilization between related organisms. This so-called inbreeding depression can reduce the ability to reproduce and adapt [53,67]. As already mentioned, our studies of small populations have shown that their diversity can be greater than that of large populations. Similar conclusions were reached by some other authors for other species [62]. This suggests that lower genetic diversity is not necessarily a criterion for small populations [62]. Similarly, Rich et al. [68] observes a large increase in diversity despite genetic drift. Treuren et al. [62] note that rare alleles occur to a greater extent in a small population. This seems to be consistent with observations in nature of species *G. nivalis* and the occurrence of their varieties that deviate from the usual description for this species.

After several years of research in the field, it has been shown that these are individual local populations, where the genetic potential of an individual species can be very high despite a relatively small population. Despite removing varieties for research and the collection from these populations to determine stability in culture, similar or exactly the same forms reappear there. The frequency of these occurrences varies greatly. Sometimes they re-occur the following year, and sometimes only after a few years. In most cases, it is in the same populations that similar or even greater deviations again occur [39,40]. Of course, there are still unexplored causes at work. Among them is the certainly the appearance of green streaks on outer perigon leaves, which appear to a greater extent after floods or after long snowy winters [57]. The appearance of some other traits can also be the result of environmental conditions. The appearance of a more pronounced scent in the cultivar *G. nivalis* ‘Dišeči Vilinec’ may be the result of late flowering. Late flowering represents competition for pollinators, whereby plants with scents draw attention to themselves more easily than those without scents.

## 4. Materials and Methods

We began searching for and collecting individual specimens of *G. nivalis* in 2000. In accordance with the permission obtained from the Ministry of the Environment, we collected specimens in various parts of Slovenia. In the wild, we collect specimens that differ in any way from the usual specimen of *G. nivalis* in terms of morphological traits. Specimens are collected in nature during bloom. Given that the time of their blooming in an individual biogeographic region or part of it depends on the temperature, we can start looking for the first blooming specimens in the coastal region as early as December. In other parts of Slovenia, the species begins blooming in January. After the coastal region, we started with a survey of populations in the locations of Goriška (western Slovenia), continued with Ljubljana and its surroundings and the pre-Alpine region, then with the Pannonian biogeographic region and the Alpine region, and finished in the Dinaric region. Surveying individual locations naturally depends on the temperature, the amount of snow, and the duration of the snow cover. The last populations of species *G. nivalis* can be surveyed at higher elevations as late as May.

**Figure 8:**
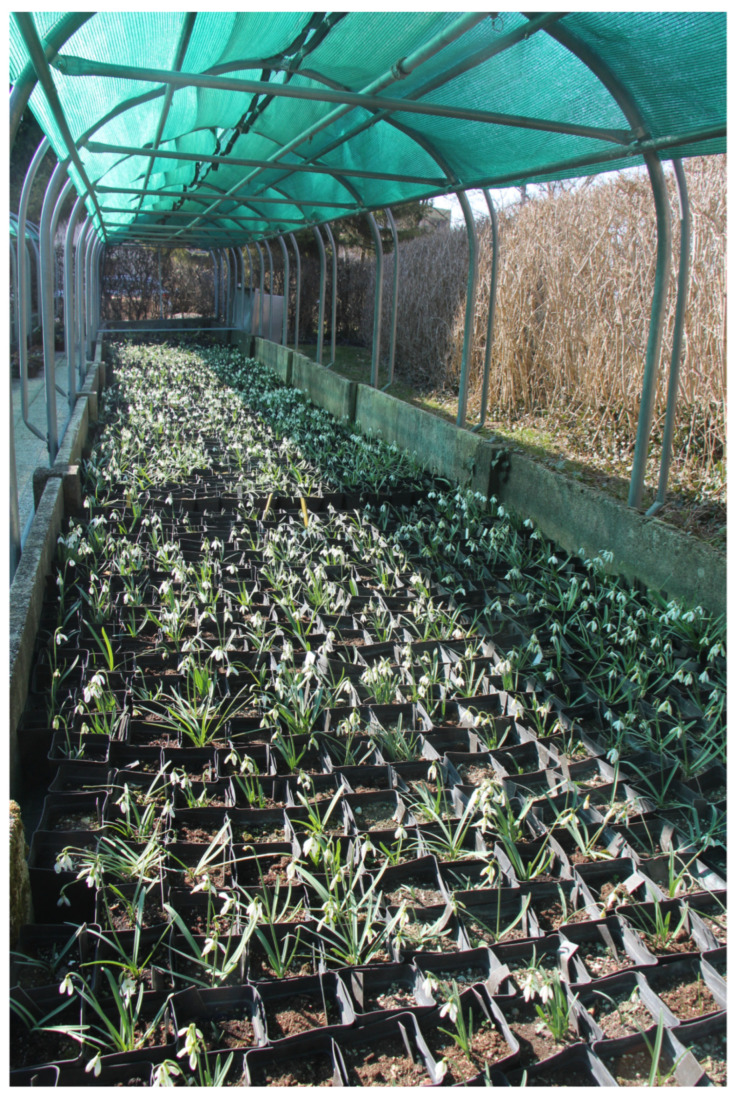
*G. nivalis* plant collection at University Botanic Gardens Ljubljana.

Localities within individual regions are chosen either randomly or on the basis of past experience, that a certain location showed high intra-species variability of *G. nivalis* in the past. At individual locations, we then systematically examine the populations and look for specimens deviating from the normal specimen. Of interest for our collection are specimens with a morphological trait that we deem potentially genetically stable based on past experience [39,44,46]. We dig up such specimens together with the bulb, place them in a PVC bag, and add a note with the date of collection and location. We also take photosgarphs of all specimens collected. The specimens are then brought to the University Botanic Gardens Ljubljana, where each bulb is planted in its own 10 × 10 cm plastic pot. For planting, we use a mixture of compost obtained from the botanic gardens, calcareous sand and sterile purchased soil with as little peat-content as possible. At the bottom of each pot we also place drainage made of volcanic stone. We then add a metal label to each pot, on which we write the year of collection, the location where the specimen was collected, and the serial number of the specimen in the collection. The pots are then placed outdoors in fenced concrete beds. Perforated plastic film is placed under the pots to prevent the growth of weeds. Shades and PVC film are installed above each of the concrete beds (Figure 8). Shading in spring and late spring months prolongs the blooming of specimens. Strong sun can cause flowers to wilt very quickly. With an extended blooming period, we have the opportunity to study individual plants in the collection for a longer period of time. Shade also reduces drying of potted soil during summer months when the bulbs are dormant. During winter months, the specimens are protected from snowfall or heavy rain with PVC foil. A sprinkle system is also installed above each of the beds, which we use to water the pots with common snowdrop.

The collection of snowdrop *G. nivalis* in the University Botanic Gardens Ljubljana currently comprises 8458 units (pots) from various biogeographical regions of Slovenia and different habitat types. As soon as the first blooming specimens appear in the collection, we begin to monitor their blooming and record our observations. Individual interesting varieties are also photo-documented. For specimens that maintain the morphological train for at least three years, we note that it is a stable form of the specimen with this trait. All varieties that are, in addition to preserving the morphological trait, also successfully divide according to the Fibonacci sequence can of course be interesting as potentially new cultivars of species *G. nivalis.* Specimens that are recognized as different from the basic morphological from of the species and whose morphological traits remain unchanged for at least three years are then compared with already existing recognized cultivars of snowdrop [18,19,21,69,70].

## Figures and Tables

**Table 1:** List of distinguishing characters of newly described varieties from already described varieties.

Cultivar Group	Comparative Cultivars	New Cultivars
**Green Group**: normal flower shape, normal number of petals, with the outer perigon leaves having a weak or strong green colour, completely coloured, or either larger or smaller spots, or with green stripes.	*G. nivalis* ‘La Boheme’ Well marked outer segments with green olive lines above apex	*G. nivalis* ‘Dišeči Vilinec’ Outer segments rounded, green marks more condensed and the segments are longer; fragrant
	*G. nivalis* ‘Bella’ Outer segments slender rounded, short merging green lines above apex, end of apex white	*G. nivalis* ‘Jakob’s Shell’ The green shape in outer segments with green shape like a shell extended till the end of apex.
	*G. nivalis* ‘Ljubljana’ The blue-green colour of outer perigon leaves spreads to the middle of segments. The stripes are condensed together.	*G. nivalis* ‘Rusalka’ The blue-green stripes are more visible and are present in first third of outer perigon leaves; flowers are larger than of ‘Ljubljana’
	*G. plicatus* ‘Castle Green Dragon’ Larger plants, inner segments totally green outer segments heavily marked with green stripes.	*G. nivalis* ‘Senožeče’ Inner segments totally green— a little les dark green. Outer segments marked with yellow green stripes, late flowering.
	*G. nivalis* ‘Carpathian Viridapicis’ Outher segments broad longitudinal, slightly pinched and pointed at apex. Apex merging green lines. The inner margin not so strong.	*Galanthus nivalis* ‘Striped Fragrant’ Outer segments similar to ‘Carpathian Viridapicis’ inner segments more visible, stronger green mark in shape of M; fragrant.
**Imperial group**: normal number of petals, but with inner perigon leaves significantly shorter than the outer ones. There is no green colouration or green spots on the outer perigon leaves, and the inner circle of perigon leaves has one green mark.	*G. plicatus* ‘Diggory’ The outer segments rounded to inside of flower. Big flower; only at the end of flowering period more opened.	*G. nivalis* ‘Cardinal’s Hat’ Very small flowers. Outer segments more condensed, rounded inside and never opened. Long flower stem.
**Skirt group**: the inner and outer segmensts more or less the same length.	*G. nivalis* skirt group or *G. nivalis* inverse group.	*G. nivalis* ‘Soldanelca’ Small flower and small plants in general. Inner and outer segments have the same length. Shape like *Soldanella minima.*

## Data Availability

Data supporting results can be found in database of University Botanic Gardens Ljubljana.
